# Multi–country analysis of routine data from integrated community case management (iCCM) programs in sub–Saharan Africa

**DOI:** 10.7189/jogh.04.020408

**Published:** 2014-12

**Authors:** Nicholas P. Oliphant, Maria Muñiz, Tanya Guenther, Theresa Diaz, Yolanda Barberá Laínez, Helen Counihan, Abigail Pratt

**Affiliations:** 1 UNICEF, Programme Division, Health, New York, USA; 2 Save the Children, Washington, DC, USA; 3 International Rescue Committee, New York, USA; 4 Malaria Consortium, London, UK; 5 Population Services International, Nairobi, Kenya

## Abstract

**Aim:**

To identify better performing iCCM programs in sub–Saharan Africa (SSA) and identify factors associated with better performance using routine data.

**Methods:**

We examined 15 evaluations or studies of integrated community case management (iCCM) programs in SSA conducted between 2008 and 2013 and with information about the program; routine data on treatments, supervision, and stockouts; and, where available, data from community health worker (CHW) surveys on supervision and stockouts. Analyses included descriptive statistics, Fisher exact test for differences in median treatment rates, the Kruskal-Wallis test for differences in the distribution of treatment rates, and Spearman’s correlation by program factors.

**Results:**

The median percent of annual expected cases treated was 27% (1–74%) for total iCCM, 37% (1–80%) for malaria, 155% (7–552%) for pneumonia, and 27% (1–74%) for diarrhoea. Seven programs had above median total iCCM treatments rates. Four programs had above median treatment rates, above median treatments per active CHW per month, and above median percent of expected cases treated. Larger populations under–five targeted were negatively associated with treatment rates for fever, malaria, diarrhea, and total iCCM. The ratio of CHWs per population was positively associated with diarrhoea treatment rates. Use of rapid diagnostic tests (RDTs) was negatively associated with treatment rates for pneumonia. Treatment rates and percent of annual expected cases treated were equivalent between programs with volunteer CHWs and programs with salaried CHWs.

**Conclusions:**

There is large variation in iCCM program performance in SSA. Four programs appear to be higher performing in terms of treatment rates, treatments per CHW per month, and percent of expected cases treated. Treatment rates for diarrhoea are lower than expected across most programmes. CHWs in many programmes are overtreating pneumonia. Programs targeting larger populations under–five tend to have lower treatment rates. The reasons for lower pneumonia treatment rates where CHWs use RDTs need to be explored. Programs with volunteer CHWs and those with salaried CHWs can achieve similar treatment rates and percent of annual expected cases treated but to do so volunteer programs must manage more CHWs per population and salaried CHWs must provide more treatments per CHW per month.

In 2010 the main causes of child mortality globally included pneumonia (18%), diarrhoea (15%), and malaria (8%), and in Africa their share was 17%, 15% and 12%, respectively [1]. In conjunction with broader efforts to address these causes of child mortality, integrated community case management (iCCM) evolved as a strategy to train, supply, and supervise community health workers (CHWs) to diagnose and treat diarrhoea, malaria, and pneumonia in communities where access to health services is poor[2]. Several studies indicate that CHWs– when appropriately trained, supplied, and supported– can effectively diagnose and treat children with uncomplicated, non–severe diarrhoea, malaria, and pneumonia [3-12]. Increasingly low and middle–income countries, including those in sub–Saharan Africa (SSA), have adopted iCCM as a component of their health strategies [13,14].

Data on iCCM program performance from routine sources– that is data collected on an ongoing basis by CHWs– provide a wealth of information but have not been fully exploited. There are few examples of analyses of routine iCCM data in the published literature [15-17]. Laínez and others reported that iCCM programs in six countries of SSA contributed to increasing the number of children treated for diarrhoea, fever, and pneumonia, but that diarrhoea treatment rates were lower than expected [15]. In Sierra Leone, they reported a strong negative correlation between treatment rates (treatments per child per year or tx/c/y) and the size of the under–five population served by CHWs and that monthly supervision of CHWs reduced the pneumonia treatment rate; the latter suggesting improved targeting of antibiotics [15]. Nsona and others reported the average monthly number of treatments per 1000 children under–five in the 28 districts of Malawi to be 20.7 for malaria (0.2 (tx/c/y), 12.9 for pneumonia (0.1 tx/c/y), and 4.6 for diarrhoea (0.0 tx/c/y), and ascribed the relative predominance of malaria treatments to the national policy of presumptive treatment of fever as malaria [16]. These studies demonstrate that routine iCCM data can provide information to improve iCCM programs early–on without having to wait for full evaluations. We build on these efforts to use routine iCCM data by broadening the geographic scope of analysis to include more programs/countries, conducting comparative analysis of iCCM treatment rates, percent of annual expected cases treated, and treatments per active CHW per month across programs/countries, and broadening the analysis of program factors associated with these parameters.

We used routine iCCM data from 2012 to answer the following questions:

1) Have certain iCCM programs in SSA performed better than others in terms of treatment rates, treatments per CHW per month, and the percent of annual expected cases treated??

2) What program factors are associated with treatment rates?

3) What program factors are associated with treatments per active CHW per month?

## METHODS

### Data sources

Twenty–three program evaluations and implementation research studies from thirteen countries in sub–Saharan Africa conducted between 2008 and 2013 were identified through a call to researchers and implementing partners [18]. The following predetermined inclusion criteria were used for our analysis: the program 1) included integrated treatment of diarrhoea, malaria, pneumonia by CHWs; 2) followed protocols for iCCM recommended by WHO/UNICEF (eg, the program did not use dual treatment for fever with antimalarials and antibiotics); 3) had at least 12 months of implementation at scale (ie, there were at least 12 months from the date at which at least 80% of deployed CHWs were trained in iCCM until the last month of routine data on treatments); and 4) had routine data available on the number of iCCM treatments provided by CHWs disaggregated by illness for 2012. Eight studies did not meet one or more of these inclusion criteria, leaving fifteen studies from ten countries for our analysis.

We obtained the following parameters from principle investigators in a standardized Microsoft Excel (2013) spreadsheet: program description, target population, routine data on treatments and stockouts of commodities based on monthly or quarterly paper–based reporting from CHWs, and routine data on supervision coverage from supervisor’s monthly or quarterly reports. Where available, additional data was provided on stockouts and/or supervision coverage from CHW surveys.

### Dependent variables

Dependent variables for our analysis included annualized treatments rates (treatments per child per year or tx/c/y) by illness, the number of treatments per active CHW per month (tx/CHW/m) by illness, and the percent of annual expected cases treated by illness in 2012 (**Table 1**). For the latter, the denominator was based on the reported population targeted by the program and regional estimates of incidence for SSA. It is recognized that in some programs not all communities would have been exposed (ie, have a CHW trained on iCCM). The percent of annual expected cases treated reflects the share of all expected cases in the entire area treated by CHWs and not only the cases from communities with CHWs trained in iCCM (ie, those with local exposure to CHWs). Similarly, household surveys typically sample across entire districts or regions (not only communities served by CHWs). In the absence of national or sub–national level estimates of incidence we used regional estimates for SSA: 0.27 for pneumonia and 3.30 for diarrhoea from Fischer Walker and others [19]; and 1.68 for malaria in average transmission areas of central Africa from Roca–Feltrer and others [20]. National level estimates of pneumonia incidence were not available for all countries in our study so we defaulted to regional level estimates for all countries [21].

**Table 1:** Dependent variable defnitions*

Indicator	Numerator	Denominator
Treatment rate (by illness)	Number of treatments (by illness) provided by CHWs in the study area over the last 12 consecutive months of the study	Number of children under five years of age in the study area
Treatments per active CHW per month	Number of treatments provided by CHWs in the last 12 consecutive months of the study/12	Number of active CHWs (the number of CHWs deployed and trained in iCCM in the study area minus attrition) during the same period
Percent of annual expected cases treated (by illness)	Number of treatments (by illness)	Annual expected number of cases (by illness) × 100

CHW – community health worker, iCCM – integrated community case management

*Annual treatment rates were asjusted by adding the product of multiplying the number of treatments by then inverse of the CHW reporting rate. For programs where CHWs do not use rapid diagnostic test (RTD), annual malaria treatment rates were adjusted downward by multiplying the number of treatments (fevers) by the RTDs positivity rate from the 2013 World Malaria Report. The adjustments above were also used for calculating the ‘Average monthly treatments per CHW’ and ‘Percent of annual expected cases treated by CHWs’. The denominator for the ‘Percent of annual expected cases treated’ is derived by multiplying the estimated average annual regional incidence by the estimated population of children under five years of age in the study area.

We adjusted the numerators (number of treatments provided by CHWs) for the three dependent variables to account for CHW reporting rates, number of months of treatment data available, and for malaria only, RDT positivity rates. To adjust for under–reporting, the number of reported treatments was adjusted upward by multiplying the number of reported treatments by the inverse of the reported CHW reporting rate. For seven of the fifteen studies, CHW reporting rates were not available so the median value (90%) from studies with available data was used.

Routine data on the number of treatments provided by CHWs were not available for the twelve months of 2012 in two of the fifteen studies despite implementing iCCM at scale during that period. Routine data were available for six months for South Sudan (SC) and seven months for Sierra Leone (UNICEF). In these cases we pro–rated the data to twelve consecutive months in 2012 based on the per month average of the available data.

For the seven programs where RDTs were not used, reported fever treatment rates were converted to malaria treatment rates for comparison purposes by adjusting the former downward using the RDT positivity rate reported for the country in the 2013 World Malaria Report [22].

### Independent variables

Other data on program factors thought to be associated with iCCM program performance (see **Figure 1** in ref. [18]) was collected from principle investigators in the Microsoft Excel spreadsheet for the programs as of 2012, including: whether CHWs charged fees for iCCM drugs or consultation; whether RDTs were used by CHWs; whether CHWs were selected by the community in which they worked; whether CHWs were paid a salary; whether implementation of the iCCM program was supported by NGOs; whether CHWs worked from a static health post (designated structure other than their home); ratio of active CHWs per 1000 children under five years of age; number of months of implementation at scale (the point in time at which at least 80% of CHWs in the field/deployed have been trained on iCCM); frequency of supervision of CHWs (as stated in policy, guidance or plans); ratio of active CHW per supervisor; percent of CHWs with no stockouts of iCCM commodities in a defined period (data was from routine sources and where available from CHW surveys; available data were for either of two periods: 1) no stockout greater than 7 days in the last 3 months or 2) no stockout of any duration in the last month; we used whichever was available; no program had both); percent of CHWs that received supervision in a defined period; population under five years of age in the program area; and the population (and percentage of) under five years of age targeted by the iCCM program in the program area.

**Figure 1:**
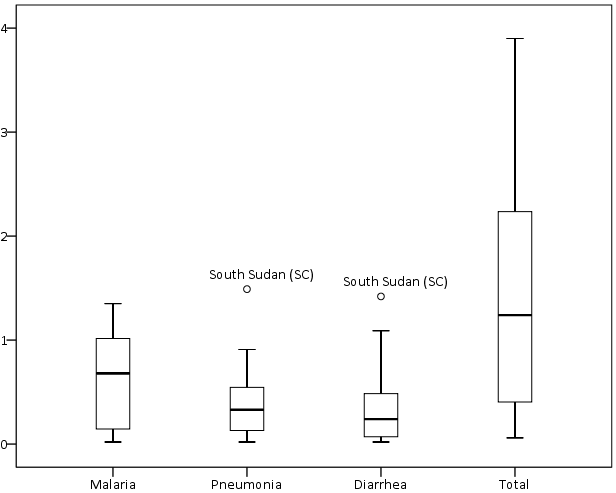
Distribution of treatment rates (treatment per child per year) by illness. SC – Save the Children.

### Analysis

We used IBM SPSS Statistics for Windows, Version 22.0 (IBM, Armonk, NY, USA) in all analyses. We calculated descriptive statistics (mean, median, range, minimum, maximum, and interquartile range) for dependent and independent variables. We tested the normality of the distribution of dependent variables using the Shapiro–Wilk test. We excluded missing values pair–wise.

To answer the question ‘Have certain iCCM programs in SSA performed better than others in terms of treatment rates, treatments per CHW per month, and the percent of annual expected cases treated?’ we calculated treatment rates, treatments per CHW per month, and the percent of annual expected cases treated by illness and study area.

To answer the question ‘What program and contextual factors are associated with treatment rates?’ we used box plots, non–parametric tests, and Spearman’s correlation to determine whether there were significant associations between independent variables and dependent variables. Non–parametric tests were used because the dependent variables were not normally distributed. We used the Kruskal-Wallis H test to determine whether there were statistically significant (*P* < 0.05) differences in the distribution of treatment rates by dichotomous independent variables. We used Fisher’s exact test, which is robust to small sample sizes and unbalanced data [23], to determine whether there were statistically significant (*P* < 0.05) differences in median treatment rates by dichotomous independent variables. We used Spearman’s rank–order correlation to test for associations between treatment rates and independent variables to complement Fisher’s exact test. We excluded outliers (values below the bottom 2% or above the upper 98% of the distribution) for the Kruskal-Wallis and Fisher’s exact tests, but we did not exclude outliers for Spearman’s correlation since it is relatively robust to outliers [24].

To answer the question ‘What program and contextual factors are associated with treatments per CHW per month?’ we undertook the same analysis described above, using treatments per active CHW per month as the dependent variable instead of treatment rates.

## RESULTS

Thirteen (87%) programs reported CHWs were selected by the community. Twelve (80%) programs reported NGOs supported the program. Six (40%) programs reported CHWs were salaried and six (40%) programs reported CHWs work at a designated post/structure in the community (the same programs that reported CHWs were salaried). Two (13%) programs reported CHWs charge fees. The mean percentage for not having stockouts was 77% (median 84%, range 34–100%) for ACTs, 78% (median 84%, range 30–100%) for antibiotics, and 81% (median 88%, range 25–100%) for ORS. Supervision coverage was 71% (median 75%, range 25–100%). There was large variation in the size of the targeted population under–five (mean 1 165 190; median 357 773; range 69 165–10.2 million), percent of the population under–five targeted (mean 76%; median 100%; range 27–100%), ratio of children under–five per active CHW (mean 328; median 94; range 35–2007), and ratio of active CHWs per 1000 children under–five (mean 10; median 11; range 1–29). There was no significant difference in the mean or median size of the targeted population under–five between programs with volunteer CHWs and salaried CHWs. However programs with volunteer CHWs had significantly higher ratios of active CHWs per 1000 children under–five compared to programs with salaried CHWs (*P* < 0.001 for mean and *P* = 0.007 for median). The data on salary, ratio of active CHW per 1000 under-fives, and designated post/structure indicate two predominant program typologies: 1) programs (n = 9) with volunteer CHWs that do not work from designated posts/structures in the community and have a larger number of CHWs per population under–five, and 2) programs (n = 6) with salaried CHWs that work at designated posts/structures in the community and have a smaller number of CHWs per population under–five (Online Supplementary Document(Online Supplementary Document), Tables s1–7).

### Treatment rates

The mean treatment rate was 0.6 (median 0.7, range 0.0–1.4) for malaria, 0.4 (median 0.3, range 0.0–1.5) for pneumonia, 0.4 (median 0.2, range 0.0–1.4) for diarrhoea, and 1.4 (median 1.2, range 0.1–3.9) for total iCCM (Online Supplementary Document(Online Supplementary Document), Table s8). Treatment rates for each illness were not normally distributed and showed high levels of variability and positive skew with outliers at the higher end of the range (**Figure 1**). Excluding outliers, we found variability less pronounced but not unimportant as per the IQRs (Online Supplementary Document(Online Supplementary Document), Table s8).

**Treatment rates varied by program area.** For example treatment rates for malaria ranged from 0.1 in Ethiopia (UNICEF) to 1.4 in Mozambique (Save the Children). However there were seven program areas (Malawi – Save the Children, Mozambique – Save the Children, Sierra Leone – International Rescue Committee, Sierra Leone – UNICEF, South Sudan – International Rescue Committee, South Sudan – Save the Children, and Uganda Central – UNICEF) that had above median total iCCM treatment rates, indicating consistency in terms of higher performing program areas and lower performing program areas (Online Supplementary Document(Online Supplementary Document), Table 9).

The reported treatment mix (the share of each illness out of the total iCCM treatment rate) did not reflect the expected treatment mix from estimated regional incidence [19,20]. The share of the total iCCM treatment rate was larger than expected for pneumonia at 30% vs 5% expected and for malaria at 43% vs 32% expected, while diarrhoea treatments represented a smaller than expected share of the treatment mix at 27% vs 63% expected.

### Treatment per active CHW per month

**Figure 2:**
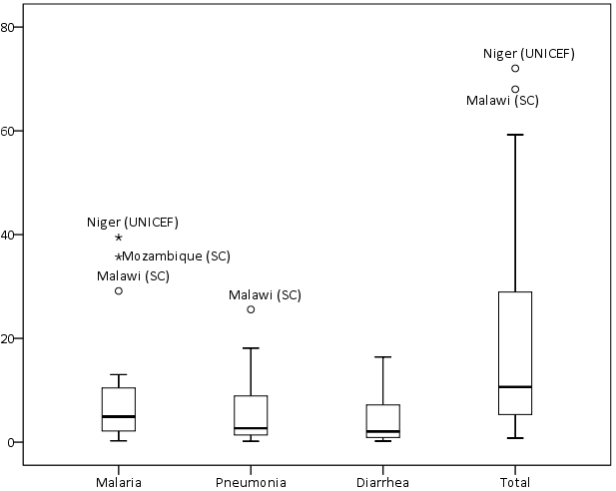
Distribution of treatments per active community health worker (CHW) per month by illness. SC – Save the Children, UNICEF – United Nation Children’s Fund.

The mean number of treatments per active CHW per month, or workload, was 10 (median 5, range 0–40) for malaria, 7 (median 3, range 0–26) for pneumonia, and 5 (median 2, range 0–16) for diarrhoea. The mean number of total iCCM treatments per active CHW per month was 22 (median 11, range 1–72). Treatments per active CHW per month were not normally distributed and showed high levels of variability and positive skew (**Figure 2**; Online Supplementary Document(Online Supplementary Document), Table s10).

Total iCCM treatments per active CHW per month varied by study area, ranging from 1 in Rwanda (IRC) and Ghana (UNICEF) to 72 in Niger (UNICEF) (Online Supplementary Document(Online Supplementary Document), Table s11). There was no significant association between program areas with above median treatments per active CHW per month and program areas with above median treatments rates.

### Percent of annual expected cases treated

**Figure 3:**
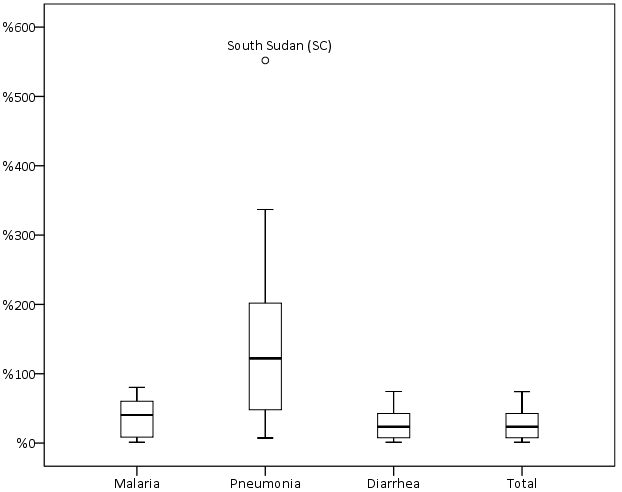
Distribution of the percent of expected cases treated by illness. SC – Save the Children.

The mean percent of expected annual cases treated was 37% (median 41%, range 1–80%) for malaria, 155% (median 122%, range 7–552%) for pneumonia, and 27% (median 14%, range 1–74%) for diarrhoea. The mean percent of annual expected cases treated for total iCCM was 27% (median 24%, range 1–74%) (Online Supplementary Document(Online Supplementary Document), Table s12). The percent of annual expected cases treated was not normally distributed for malaria or pneumonia and showed high levels of variability and positive skew (**Figure 3**; Online Supplementary Document(Online Supplementary Document), Table s12).

The mean percent of expected annual cases treated for total iCCM varied by study area, ranging from 1% in Ethiopia (UNICEF) to 74% in South Sudan (Save the Children). Variation was greatest for pneumonia which ranged from 1% in Ethiopia (UNICEF) to 552% in South Sudan (Save the Children) (Online Supplementary Document(Online Supplementary Document), Table s13).

### Program and contextual factors associated with treatment rates

Programs with larger targeted populations under–five had significantly less variation in treatment rates and lower median treatment rates for fever (*P* = 0.041), malaria (*P* = 0.048), and diarrhea (*P* = 0.041), and spearman’s correlation indicated significant negative associations for fever, malaria, diarrhea, and total iCCM. We found a similar pattern for pneumonia but the difference in medians was not statistically significant. Programs that reported CHWs use RDTs had similar median treatment rates for pneumonia but significantly less variation (*P* = 0.042) compared to programs that reported CHWs do not use RDTs, and spearman’s correlation indicated a significant negative association (*P* = 0.037). We found a similar but insignificant pattern between RDTs and fever treatment rates (**Table 2**). Spearman’s correlation indicated a significant positive association between the continuous variable for the ratio of active CHWs per 1000 under-fives and the treatment rate for diarrhoea (Online Supplementary Document(Online Supplementary Document), Table s14).

**Table 2:** Tests for differences in the distribution and median of treatment rates and tests of asscociation by independent variables*

Factor		Fever	Malaria	Pneumonia	Diarrhea	Total
**Above median population U5 targeted:**						
No (No.)	1.76 (8)	1.03 (4)	1.03 (4)	0.41 (8)	0.49 (8)	2.24 (8)
Yes (No.)	0.33 (7)	0.10 (6)	0.10 (6)	0.28 (7)	0.12 (7)	0.56 (7)
Median test	0.041†	0.048†	0.048	0.315	0.041†	0.132
Distribution test	0.041†	0.019†	0.019	0.105	0.028†	0.041†
Association test	0.031†	0.014†	0.014	0.106	0.021†	0.011†
**Above median ratio of active CHWs per 1000 U5s:**						
No (No.)	0.98 (8)	0.64 (8)	0.64 (8)	0.34 (8)	0.20 (8)	1.05 (8)
Yes (No.)	1.72 (7)	0.83 (7)	0.83 (7)	0.31 (6)	0.46 (7)	1.81 (7)
Median test	0.619	0.619	0.619	1.000	0.619	0.619
Distribution test	0.728	0.908	0.908	0.948	0.298	0.562
Association test	0.742	0.913	0.913	0.700	0.315‡	0.582
**RDTs:**						
No (No.)	1.92 (7)	NA	NA	0.64 (7)	0.24 (7)	2.03 (7)
Yes (No.)	0.98 (8)	NA	NA	0.23 (8)	0.19 (8)	1.05 (8)
Median test	0.619	NA	NA	0.132	1.000	0.619
Distribution test	0.165	NA	NA	0.042†	0.298	0.224
Association test	0.173	NA	NA	0.037†	0.315	0.237

U5 – childern under five, CHW – community health worker, RDT – rapid diagnostic test, NA – not applicable

*Where the sum of “Yes” and “No” does not equal 15, outliers have been removed from the analysis. Fisher’s exact test was used to test diffference in median. Kruskal-Wallis test was used to test difference in distributions. Spearman’s ρ was used to test for associations.

†Significant (*P* < 0.05).

‡Association with continous variable for the ration of active CHWs per 1000 U5 was marginally insignificant (*P* = 0.060).

We found no significant differences in median treatment rates and the following factors: NGOs supported implementation, CHWs worked at a fixed post, CHWs were salaried, CHWs were selected by the community, above median ratio of active CHW per 1000 under-fives, supervision was meant to be monthly by plan or policy, above median ratio of active CHWs per supervisor; above median supervision coverage, above median percent of CHWs with no stockout of antibiotics, above median percent of CHWs with no stockouts of ACTs, above median percent of CHWs with no stockouts of ORS, above median percent of the population under–five targeted, active case finding, or fees. Spearman’s correlation corroborated the results from Fisher’s exact tests (Online Supplementary Document(Online Supplementary Document), Table s14). We found no difference (*P* = 0.552) in median treatment rates between program typology 1 (volunteer CHWs that do not work from a designated post/structure and serve larger populations under–five) and program typology 2 (salaried CHWs that work from a designated post/structure and serve smaller populations under–five).

### Program and contextual factors associated with treatments per active CHW per month

We found significantly higher median treatments per active CHW per month for programs with salaried CHWs (*P* = 0.041) and for programs with CHWs that work at a designated post/structure (*P* = 0.041), and found perfect collinearity between programs with these two characteristics. Similarly we found significantly higher treatments per active CHW per month among program typology 2 compared to program typology 1. No significant differences in medians were found between total iCCM treatments per active CHW per month and the following factors: NGOs supported implementation, supervision was meant to be monthly by plan or policy, above median ratio of active CHWs per supervisor, above median supervision coverage, above median no stockouts of ACTs, above median no stockouts of antibiotics, above median no stockouts of ORS, or active case finding. Spearman’s correlation corroborated all results from Fisher’s exact test, except for the following instances. Spearman’s correlation indicated significant negative associations between treatments per active CHW per month for all illnesses and fees for iCCM, between treatments per active CHW per month for all illnesses and the percent of the under–five population targeted, and between pneumonia treatments per active CHW per month and whether CHWs were selected by the community (Online Supplementary Document(Online Supplementary Document), Table s15).

## DISCUSSION

Our analysis indicated large variation in performance of iCCM programs. We identified higher performing programs, lower performing programs, and factors associated with performance using routine data. Our results for treatment rates were consistent with others studies [15,17] and for treatments per active CHW per month [16,25,26]. In half of the fifteen studies CHWs treated 24% or fewer of the total annual expected cases, and in four programs CHWs treated 5% or less of total annual expected cases. This is cause for concern but an opportunity to learn.

Programs with above median treatment rates and above median percent of annual expected cases treated were consistent across illnesses. We found no association between program areas with above median total iCCM treatment rate and program areas with above median number of treatments per active CHW per month, and we found differences in the factors associated with each. We found four program areas (Malawi – Save the Children, Mozambique – Save the Children, South Sudan – Save the Children, and Uganda Central – UNICEF) that had above median treatment rates, above median treatments per active CHW per month, and above median percent of expected cases treated.

Our results indicate that diarrhoea treatment rates and the percent of annual expected cases treated for diarrhoea are lower than expected. Similar results were reported by Laínez and others [15] and by Mugani and others [17]. Laínez and others contend that where CCM for diarrhoea was added to CCM for malaria (eg, Rwanda) a focus on malaria may persist and overshadow diarrhoea, resulting in lower treatment rates [15]. Only 20% of CHWs treating diarrhoea reported stockouts of ORS across the program areas, and while the stockout data do not reflect trends or the dynamics of stockouts one has to question whether demand–side factors were addressed. The literature suggests that managers must address a number of demand–side factors including caregiver’s perceptions of diarrhoea, their perceptions of the effectiveness of ORS, and preferences for traditional remedies [27-32].

Our results indicate CHWs in most programs areas are overtreating pneumonia, and in several program areas they are doing so by large orders of magnitude. Similar results were reported by Mugani and others [17]. The appropriateness of the regional estimates of pneumonia incidence may be questioned, however the magnitude of the difference between our pneumonia treatment rates and what would be expected suggests overtreatment is the more likely explanation. In addition our results for fever/malaria and diarrhoea seem plausible, lending further support to this conclusion. A recent review of pneumonia treatment by CHWs in SSA concluded that CHWs may be overtreating [33] however overtreatment has also been reported at facility level [34,35]. We found a significant negative association between the use of RDTs and pneumonia treatment rates and less variation among programs using RDTs. The mechanisms driving this association were not clear. We did not capture data on whether CHWs used respiratory rate timers (RRTs). It is possible that where CHWs used RDTs they may have also used RRTs, and that use of RRTs may have improved targeting of antibiotic treatment.

We found significant negative associations between size of the targeted population under-five and treatment rates, but more variation in treatment rates among program areas targeting smaller under-five populations. Higher treatment rates among programs targeting smaller under-five populations (eg, a district or portion thereof) may be due to the ability to concentrate resources. It is unclear why there was greater variation in treatment rates for programs targeting smaller under-five populations.

We found a significant positive association between the ratio of active CHWs per 1000 children under-five and the diarrhoea treatment rate; however the association was driven entirely by four program areas in two countries (Sierra Leone – International Rescue Committee, Sierra Leone – UNICEF, South Sudan – International Rescue Committee, and South Sudan – Save the Children). Laínez and others found a similar association with the total iCCM treatment rate in Sierra Leone [15]. It is not clear why this association might be present in these outlier programs for diarrhoea but not for malaria or pneumonia.

We found that factors associated with treatments per active CHW per month differed from factors associated with treatment rates. Unlike for treatment rates, we found a significant positive association between whether CHWs were salaried and the number of total iCCM treatments per active CHW per month. In programs with salaried CHWs, CHWs worked from designated posts/structures in the community, and the programs tended to have below median ratios of CHWs per 1000 children under–five targeted. This indicated two interesting program typologies: programs with fewer CHWs per population under-five who work from designated posts/structures in the community and are salaried, and programs with more CHWs per population under-five who do not work from designated posts/structures and are volunteers. The data indicate these two types of programs achieved equivalent treatment rates, but to do so programs with salaried CHWs treated more cases per active CHW and programs with volunteers managed more CHWs per population under-five.

There are several limitations to our study. Selection bias is a possibility since we only drew from studies we were made aware of through the call to researchers and implementing partners and may have missed other programs with data that could have contributed to this analysis. However we included data from several larger iCCM programs which may make our results reasonably representative. The small number of study areas and the point–in–time nature of this data may have decreased our ability to detect robust associations between our dependent variables and independent variables. While Fisher’s exact test is robust to small sample sizes and unbalanced data, a larger sample size may have revealed greater variation and may have allowed for a wider range of robust analytical methods including multivariate analysis. We were also limited by data collected by the study teams and there are factors of interest for which we did not have data (eg, information on demand generating activities, data on referral of severe cases, aggregate data on caregiver’s knowledge and socioeconomic status). The bivariate associations between performance and program/contextual factors do not control for confounding factors, nor do they consider interactive effects (ie, effect modifiers). The static view provided by our analysis may conceal dynamic relationships (eg, for stockouts). Given the above, our results on the associations between performance and program/contextual factors should be interpreted with caution. Our analysis is based on routine data reported to us by principle investigators of each study and we were unable to assess the quality of the reported data. Principle investigators reported the targeted population under–five, thus our analysis is based on the reported targeted population under–five rather than the actual age groups targeted by CHWs (eg, 2–59 months). However this should not affect the comparisons across countries or the analysis of associations since the percentage of younger groups such as 0–2 months not targeted by CHWs should be similar across countries. CHW reporting rates were not available for seven of fifteen studies and had to be imputed. In two of the fifteen studies, routine data on treatments provided by CHWs were not available for each of the last twelve consecutive months of the study and values had to be imputed. In seven of fifteen studies that reported CHWs do not use RDTs we used national RDT positivity rates in the absence of local data representative of study areas to adjust fever treatments downward to be comparable with reported malaria treatments from studies that reported CHWs use RDTs. Our analysis used regional estimates of incidence in the absence of national estimates for each illness and all countries. Although we expanded the geographic scope of previous analysis using routine data and revealed variation in iCCM treatment rates and percent of annual expected cases treated across program areas, our analysis may mask inequities within program areas. Additionally, our analysis did not consider quality and timeliness of treatment– key factors if iCCM is to have an impact on child health.

Despite these limitations our analysis has provided new insights about iCCM programs in SSA, demonstrating the value of leveraging routine data. More research is needed to understand the drivers of variation in treatment rates and the percent of annual expected cases treated but our analysis points to promising leads. The underlying mechanisms driving lower diarrhoea treatment rates urgently need to be identified and addressed, and that CHWs in many program areas are over–treating pneumonia is also of concern and needs to be addressed.
