# Supplementary material for: Multi–country analysis of routine data from integrated community case management (iCCM) programs in sub–Saharan Africa
Source: J Glob Health. 2014 Dec;4(2):020408. doi: 10.7189/jogh.04.020408 (PMC4267092; doi:10.7189/jogh.04.020408)
Supplement: Online Supplementary Document [file jogh-04-020408-s001.pdf]

Online Supplementary Document

Oliphant et al. Multi-country analysis of routine data from integrated community case management programs in sub-Saharan Africa

J Glob Health 2014;4:020408

Supplementary Table s1. Descriptive statistics for policy variables

		CHW receives a salary	CHWs selected by the community	RDTs used by CHWs	Fees charged for iCCM	Active case finding
N	Valid	15	15	15	15	15
	Missing	0	0	0	0	0
Mean		.40	.87	.53	.13	.07
Sum		6	13	8	2	1

Supplementary Table s2. Descriptive statistics for deployment variables

		Program supported by an NGO	CHWs work at a fixed/designated structure	Ratio of active CHWs per 1,000 U5s in study area	Ratio of targeted U5s per active CHW in study area
N	Valid	15	15	15	15
	Missing	0	0	0	0
Mean		.80	.40	10.4	327.7
Median				10.7	93.5
Range				28.4	1972.4
Minimum				0.5	34.6
Maximum				28.9	2007.0
Sum		12	6	156.0	4914.8
Percentiles	25			2.6	59.8
	50			10.7	93.5
	75			16.7	385.0

Supplementary Table s3. Descriptive statistics for supply variables

		Percent of CHWs with no stockout of ACTs in a defined period (last month or longer than 7 days in the last 3 months) (routine or survey)	Percent of CHWs with no stockout of Antibiotics in a defined period (last month or longer than 7 days in the last 3 months) (routine or survey)	Percent of CHWs with no stockout of ORS in a defined period (last month or longer than 7 days in the last 3 months) (routine or survey)
N	Valid	14	14	15
	Missing	1	1	0
Mean		77.4	81.2	79.6
Median		84.0	87.5	88.0
Range		66.0	66.0	75.0
Minimum		34.0	34.0	25.0
Maximum		100.0	100.0	100.0
Percentiles	25	69.3	69.8	77.0
	50	84.0	87.5	88.0
	75	89.0	94.8	94.0

Supplementary Table s4. Descriptive statistics for routine supervision coverage

Supervision coverage (routine)		
N	Valid	13
	Missing	2
Mean		71.2
Median		75.0
Range		75.0
Minimum		25.0
Maximum		100.0
Percentiles	25	46.0
	50	75.0
	75	95.0

Supplementary Table s5. Descriptive statistics for context variables

		Population under 5 targeted (in last year of treatment data)	Percent of population under 5 targeted (in last year of treatment data)
N	Valid	15	15
	Missing	0	0
Mean		1,165,190	75.9
Median		343,087	100.0
Range		10,161,285	73.0
Minimum		69,165	27.0
Maximum		10,230,450	100.0
Percentiles	25	103,017	46.0
	50	343,087	100.0
	75	1,210,691	100.0

Supplementary Table s6. Association between size of the targeted population under-five and salaried CHWs

Group Statistics

CHW receives a salary		N	Mean	Std. Deviation	Std. Error Mean
Population under 5 targeted (in last year of treatment data)	No	9	355,785	380,862	126,954
	Yes	6	2,379,296	3,901,749	1,592,882

Independent Samples Test

		Levene's Test		t-test for Equality of Means						
									95% Confidence	
		F	Sig.	t	df	Sig. (2-tailed)	Mean Difference	Std. Error Difference	Lower	Upper
Population under 5 targeted in study area (in last year of treatment data)	Equal variances assumed	7.177	.019	-1.575	13	.139	-2023510.7	1285011.1	-4799608.5	752587.1
	Equal variances not assumed			-1.266	5.064	.261	-2023510.7	1597933.5	-6115660.9	2068639.6

Correlations

			Population under 5 targeted (in last year of treatment data)	CHW receives a salary
Spearman's rho	Population under 5 targeted (in last year of treatment data)	Correlation Coefficient	1.000	.472
		Sig. (2-tailed)		.075
		N	15	15
	CHW receives a salary	Correlation Coefficient	.472	1.000
		Sig. (2-tailed)	.075	
		N	15	15

Supplementary Table s7. Association between ratio of active CHWs per 1,000 U5s and salaried CHWs

Group Statistics					
CHW receives a salary		N	Mean	Std. Deviation	Std. Error Mean
Ratio of active CHWs per 1,000 U5s in study area	No	9	15.9	6.3	2.1
	Yes	6	2.1	1.0	0.4

Independent Samples Test										
		Levene's Test for Equality of Variances		t-test for Equality of Means						
									95% Confidence Interval of the Difference	
		F	Sig.	t	df	Sig. (2-tailed)	Mean Difference	Std. Error Difference	Lower	Upper
Ratio of active CHWs per 1,000 U5s in study area	Equal variances assumed	5.910	.030	5.289	13	.000	13.8	2.6	8.2	19.5
	Equal variances not assumed			6.488	8.616	.000	13.8	2.1	9.0	18.7

Correlations				
			CHW receives a salary	Ratio of active CHWs per 1,000 U5s in study area
Spearman's rho	CHW receives a salary	Correlation Coefficient	1.000	-.850**
		Sig. (2-tailed)		.000
		N	15	15
	Ratio of active CHWs per 1,000 U5s in study area	Correlation Coefficient	-.850**	1.000
		Sig. (2-tailed)	.000	
		N	15	15

Supplementary Table 8. Distribution of treatment rates by (treatments per child per year) by illness

		Fever treatments per child per year (adjusted for CHW reporting rates)	Malaria treatments per child per year (adjusted for CHW reporting rates)	Pneumonia (not adjusted for FB) treatments per child per year (adjutsted for CHW reporting rates)	Diarrhea treatments per child per year (adjusted for CHW reporting rates)	Total iCCM treatments per child per year (pneumonia not adjusted for FB; adjusted for CHW reporting rates)
N	Valid	15	15	15	15	15
	Missing	0	0	0	0	0
Mean		1.1	0.6	0.4	0.4	1.4
Median		1.1	0.7	0.3	0.2	1.2
Range		2.3	1.3	1.5	1.4	3.8
Minimum		0.0	0.0	0.0	0.0	0.1
Maximum		2.4	1.4	1.5	1.4	3.9
Percentiles	25	0.2	0.1	0.1	0.1	0.3
	50	1.1	0.7	0.3	0.2	1.2
	75	1.9	1.0	0.6	0.5	2.2
Shapiro-Wilk	Statistic	0.889	0.884	0.864	0.818	0.920
	Sig.	0.064	0.055	0.027	0.006	0.194

Note: All rates adjusted for CHW reporting rates; malaria rates adjusted for RDT positivity in countries not using RDTs; total iCCM is for malaria, pneumonia, and diarrhea.

Supplementary Table s9. Treatment rates (treatments per child per year) by illness and study (ordered from highest to lowest)

Malaria		Pneumonia		Diarrhea		Total	
Mozambique (SC)	1.4	South Sudan (SC)	1.5	South Sudan (SC)	1.4	South Sudan (SC)	3.9
Sierra Leone (IRC)	1.1	Malawi (SC)	0.9	South Sudan (IRC)	1.1	South Sudan (IRC)	2.6
Malawi (SC)	1.0	Uganda Central (UNICEF)	0.8	Sierra Leone (IRC)	0.7	Malawi (SC)	2.4
Sierra Leone (UNICEF)	1.0	South Sudan (IRC)	0.6	Mozambique (SC)	0.5	Mozambique (SC)	2.2
Uganda Central (UNICEF)	1.0	Sierra Leone (IRC)	0.5	Malawi (SC)	0.5	Sierra Leone (IRC)	2.2
South Sudan (SC)	1.0	Malawi (UNICEF)	0.4	Sierra Leone (UNICEF)	0.5	Uganda Central (UNICEF)	2.0
South Sudan (IRC)	0.8	Mozambique (SC)	0.4	Niger (UNICEF)	0.3	Sierra Leone (UNICEF)	1.8
Niger (UNICEF)	0.7	Sierra Leone (UNICEF)	0.3	Uganda Central (UNICEF)	0.2	Niger (UNICEF)	1.2
Zambia (MC)	0.6	Niger (UNICEF)	0.3	Malawi (UNICEF)	0.2	Malawi (UNICEF)	0.9
Malawi (UNICEF)	0.3	Uganda West (MC)	0.3	Uganda West (MC)	0.1	Zambia (MC)	0.9
Uganda West (MC)	0.2	Zambia (MC)	0.2	Zambia (MC)	0.1	Uganda West (MC)	0.6
Rwanda (IRC)	0.1	Rwanda (IRC)	0.1	Ghana (UNICEF)	0.1	Rwanda (IRC)	0.3
Ghana (UNICEF)	0.0	Ghana (UNICEF)	0.0	Rwanda (IRC)	0.0	Ghana (UNICEF)	0.1
Mozambique (UNICEF)	0.0	Ethiopia (UNICEF)	0.0	Mozambique (UNICEF)	0.0	Mozambique (UNICEF)	0.1
Ethiopia (UNICEF)	0.0	Mozambique (UNICEF)	0.0	Ethiopia (UNICEF)	0.0	Ethiopia (UNICEF)	0.1
Note: All rates adjusted for CHW reporting rates; malaria rates adjusted for RDT positivity in countries not using RDTs; total iCCM is for malaria, pneumonia, and diarrhea.							

Supplementary Table s10. Distribution of treatments per CHW per month by illness

		Fever	Malaria	Pneumonia	Diarrhea	Total
N	Valid	15	15	15	15	15
	Missing	0	0	0	0	0
Mean		15.3	10.4	6.6	5.0	22.0
Median		6.1	4.9	2.7	2.1	10.6
Range		65.7	39.2	25.4	16.2	71.2
Minimum		0.5	0.3	0.2	0.2	0.8
Maximum		66.2	39.5	25.6	16.4	72.0
Percentiles	25	2.4	1.2	1.3	0.9	4.2
	50	6.1	4.9	2.7	2.1	10.6
	75	29.6	13.0	10.4	7.3	38.4
Shapiro-Wilk	Statistic	0.774	0.726	0.822	0.820	0.774
	Sig.	0.002	0.000	0.007	0.007	0.002

Note: All adjusted for CHW reporting rates; malaria adjusted for RDT positivity in countries not using RDTs; total iCCM is for malaria, pneumonia, and diarrhea.

Supplementary Table s11. Treatments per CHW per month by illness and study (ordered from highest to lowest)

Malaria		Pneumonia		Diarrhea		Total	
Niger (UNICEF)	39	Malawi (SC)	26	Niger (UNICEF)	16	Niger (UNICEF)	72
Mozambique (SC)	36	Malawi (UNICEF)	18	Malawi (SC)	13	Malawi (SC)	68
Malawi (SC)	29	Niger (UNICEF)	16	Mozambique (SC)	13	Mozambique (SC)	59
Malawi (UNICEF)	13	Mozambique (SC)	10	Malawi (UNICEF)	7	Malawi (UNICEF)	38
Uganda Central (UNICEF)	8	South Sudan (SC)	7	South Sudan (SC)	7	South Sudan (SC)	19
Zambia (MC)	6	Uganda Central (UNICEF)	6	South Sudan (IRC)	5	Uganda Central (UNICEF)	16
Mozambique (UNICEF)	6	Mozambique (UNICEF)	4	Mozambique (UNICEF)	4	Mozambique (UNICEF)	14
South Sudan (SC)	5	South Sudan (IRC)	3	Sierra Leone (UNICEF)	2	South Sudan (IRC)	11
Sierra Leone (UNICEF)	5	Uganda West (MC)	2	Sierra Leone (IRC)	2	Zambia (MC)	9
South Sudan (IRC)	3	Zambia (MC)	2	Uganda Central (UNICEF)	2	Sierra Leone (UNICEF)	8
Sierra Leone (IRC)	3	Sierra Leone (UNICEF)	1	Zambia (MC)	1	Sierra Leone (IRC)	6
Uganda West (MC)	1	Sierra Leone (IRC)	1	Uganda West (MC)	1	Uganda West (MC)	4
Rwanda (IRC)	1	Ethiopia (UNICEF)	1	Ethiopia (UNICEF)	1	Ethiopia (UNICEF)	2
Ethiopia (UNICEF)	1	Rwanda (IRC)	0	Ghana (UNICEF)	0	Rwanda (IRC)	1
Ghana (UNICEF)	0	Ghana (UNICEF)	0	Rwanda (IRC)	0	Ghana (UNICEF)	1
Note: All adjusted for CHW reporting rates; malaria adjusted for RDT positivity in countries not using RDTs; total iCCM is for malaria, pneumonia, and diarrhea.							

Supplementary Table s12. Distribution of the percent of expected cases treated by illness

		Malaria	Pneumonia	Diarrhea	Total
N	Valid	15	15	15	15
	Missing	0	0	0	0
Mean		36.7	155.3	27.0	26.9
Median		40.5	122.2	23.6	23.6
Range		79.2	544.5	73.2	73.1
Minimum		1.2	7.4	1.1	1.1
Maximum		80.4	551.9	74.3	74.3
Percentiles	25	7.7	29.6	4.8	4.8
	50	40.5	122.2	23.6	23.6
	75	60.7	237.0	42.7	42.7
Shapiro-Wilk	Statistic	.884	.864	.921	.920
	Sig.	.055	.027	.196	.194
Note: All adjusted for CHW reporting rates; malaria adjusted for RDT positivity in countries not using RDTs; total iCCM is for malaria, pneumonia, and diarrhea.					

Supplementary Table s13. Percent of expected cases treated by illness and study (ordered from highest to lowest)

Malaria		Pneumonia		Diarrhea		Total	
Mozambique (SC)	80	South Sudan (SC)	552	South Sudan (SC)	74	South Sudan (SC)	74
Sierra Leone (IRC)	64	Malawi (SC)	337	South Sudan (IRC)	49	South Sudan (IRC)	49
Malawi (SC)	62	Uganda Central (UNICEF)	289	Malawi (SC)	46	Malawi (SC)	46
Sierra Leone (UNICEF)	61	South Sudan (IRC)	237	Mozambique (SC)	43	Mozambique (SC)	43
Uganda Central (UNICEF)	60	Sierra Leone (IRC)	167	Sierra Leone (IRC)	43	Sierra Leone (IRC)	42
South Sudan (SC)	59	Malawi (UNICEF)	152	Uganda Central (UNICEF)	39	Uganda Central (UNICEF)	39
South Sudan (IRC)	49	Mozambique (SC)	144	Sierra Leone (UNICEF)	35	Sierra Leone (UNICEF)	34
Niger (UNICEF)	41	Sierra Leone (UNICEF)	122	Niger (UNICEF)	24	Niger (UNICEF)	24
Zambia (MC)	35	Niger (UNICEF)	104	Malawi (UNICEF)	16	Malawi (UNICEF)	16
Malawi (UNICEF)	17	Uganda West (MC)	104	Zambia (MC)	16	Zambia (MC)	16
Uganda West (MC)	10	Zambia (MC)	67	Uganda West (MC)	11	Uganda West (MC)	11
Rwanda (IRC)	8	Rwanda (IRC)	30	Rwanda (IRC)	5	Rwanda (IRC)	5
Ghana (UNICEF)	2	Ghana (UNICEF)	11	Ghana (UNICEF)	2	Ghana (UNICEF)	2
Mozambique (UNICEF)	2	Ethiopia (UNICEF)	7	Mozambique (UNICEF)	2	Mozambique (UNICEF)	2
Ethiopia (UNICEF)	1	Mozambique (UNICEF)	7	Ethiopia (UNICEF)	1	Ethiopia (UNICEF)	1

Note: All adjusted for CHW reporting rates; malaria adjusted for RDT positivity in countries not using RDTs; total iCCM is for malaria, pneumonia, and diarrhea.
